# Impact of genetic polymorphisms on chemotherapy toxicity in childhood acute lymphoblastic leukemia

**DOI:** 10.3389/fgene.2012.00249

**Published:** 2012-11-22

**Authors:** Guillermo Gervasini, Jose M. Vagace

**Affiliations:** ^1^Department of Medical and Surgical Therapeutics, Division of Pharmacology, Medical School, University of ExtremaduraBadajoz, Spain; ^2^Service of Pediatric Hematology, University Hospital Infanta CristinaBadajoz, Spain

**Keywords:** acute lymphoblastic leukemia, pharmacogenetics, toxicity, chemotherapy, genetic polymorphisms

## Abstract

The efficacy of chemotherapy in pediatric acute lymphoblastic leukemia (ALL) patients has significantly increased in the last 20 years; as a result, the focus of research is slowly shifting from trying to increase survival rates to reduce chemotherapy-related toxicity. At the present time, the cornerstone of therapy for ALL is still formed by a reduced number of drugs with a highly toxic profile. In recent years, a number of genetic polymorphisms have been identified that can play a significant role in modifying the pharmacokinetics and pharmacodynamics of these drugs. The best example is that of the *TPMT* gene, whose genotyping is being incorporated to clinical practice in order to individualize doses of mercaptopurine. However, there are additional genes that are relevant for the metabolism, activity, and/or transport of other chemotherapy drugs that are widely use in ALL, such as methotrexate, cyclophosphamide, vincristine, L-asparaginase, etoposide, cytarabine, or cytotoxic antibiotics. These genes can also be affected by genetic alterations that could therefore have clinical consequences. In this review we will discuss recent data on this field, with special focus on those polymorphisms that could be used in clinical practice to tailor chemotherapy for ALL in order to reduce the occurrence of serious adverse effects.

## INTRODUCTION

In the last 20 years, the chemotherapy treatment of pediatric acute lymphoblastic leukemia (ALL), the most common malignancy in children, has reached success rates of up to 90%. This achievement has allowed limiting the administration of damaging cranial irradiation to rare cases with central nervous system (CNS) infiltration (Laningham et al., 2007). However, the clinical routine of chemotherapy treatments in these patients includes intrathecal and high intravenous doses of various drugs with a highly toxic profile. This is therefore a source of a wide variety of complications that add up to those caused by peripheral blood cell depression or the disease itself (Vagace and Gervasini, 2011). In fact, the toxicity of chemotherapy is a common cause of morbidity and mortality in children with ALL, as well as a frequent source of sequelae at mid-long term. These adverse effects are often the consequence of direct toxicity in healthy tissue, as a result of the low specificity displayed by these drugs and become more frequent as the treatment is intensified. In particular, central neurotoxicity is a major clinical concern in pediatric patients (Vagace et al., 2012).

In this scenario, it is obvious that any factor able to modify either the pharmacokinetics or pharmacodynamics of chemotherapy drugs holds the potential to be critical for the occurrence of serious adverse effects in ALL patients. One such factor is the presence of genetic polymorphisms in genes coding for drug-metabolizing enzymes, transporters, or drug targets. Genetic association studies in this field have traditionally been focused on efficacy parameters in general and survival in particular and therefore the body of work on the association with toxicity is still scarce.

In this review we will summarize what is currently known about how genetic variability can affect the toxicity induced by the main drugs used in the chemotherapy of ALL (see summary in **Table 1**). A special focus will be put on the discussion on those genetic analyses with the potential to tailor chemotherapy regimes in the ALL setting.

**Table 1:** Summary of genes and polymorphisms with a putative relevant role in chemotherapy-induced toxicity in acute lymphoblastic leukemia.

Gene	Polymorphism	Drug affected; main effect	Reference
*ABCB1*	C3435T, G2677T/A, C1236T	IMT^a^; Less common toxicity-related dose reduction	Gurney et al. (2007)
*CYP2B6*	*2A, *4	CFD^b^; Hemorrhagic cystitis, oral mucositis	Rocha et al. (2009)
*DCK*	Several SNPs	Ara-C^c^; higher susceptibility to drug effects	Hartford et al. (2009)
*DHFR*	19-bp deletion	MTX^d^, hepatotoxicity	Ongaro et al. (2009)
*GRIA1*	Several SNPs	ASP; higher risk of hypersensitivity	Chen et al. (2010)
*ITPA*	c.94C > A	6-MP; fever, hepatotoxicity	Stocco et al. (2009), Wan Rosalina et al. (2011)
*ITPA*	IVS + 21A > C	6-MP; higher risk of myelotoxicity	Hawwa et al. (2008)
*MTHFR*	C677T	MTX; neurotoxicity, hepatotoxicity, myelosuppression	Strunk et al. (2003), Mahadeo et al. (2010), D’Angelo et al. (2011), Yang et al. (2012)
*MTHFR*	A1298C	MTX^e^, hematological toxicity	Kantar et al. (2009)
*MTRR*	A66G	MTX, oral mucositis	Huang et al. (2008)
*RFC1*	G-80A	MTX, overall toxicity	Shimasaki et al. (2006), Imanishi et al. (2007)
*TPMT*	*2, *3A, *3C	6-MP; Acute hematopoietic toxic effects	McLeod et al. (1999), Weinshilboum (2001), Pui et al. (2004)

a Only data on chronic myeloid leukemia patients are available

b Several types of leukemia were included in the study

c Preliminary data on cell lines

d Adult patients

e Controversial association

*6-MP, 6-mercaptopurine; MTX, methotrexate; ASP, L-asparaginase; CFD, cyclophosphamide; Ara-C, cytosine arabinoside; IMT, imatinib*

## L-ASPARAGINASE

L-asparaginase is a standard component in the initial treatment of childhood ALL which induces the depletion of the essential amino acid L-asparagine in the tumor cells resulting in inhibition of protein synthesis.

This drug has been related to serious adverse effects in ALL patients, such as acute pancreatitis (Flores-Calderon et al., 2009) or cerebrovascular accidents (Gugliotta et al., 1992). In addition, hypersensitivity reactions occur in up to 25% of the patients and 10% of those may experience life-threatening anaphylactic reactions (Cortijo-Cascajares et al., 2012). Studies on the pharmacogenetics of asparaginase in ALL are in their early stages. A large study recently carried out at St Jude Children’s Hospital by Chen et al. (2010) interrogated more than 500 000 single nucleotide polymorphisms (SNPs) in 485 children with ALL and found that five mutations (rs4958351, rs10070447, rs6890057, rs4958676, and rs6889909) in the *GRIA1* gene were associated with the occurrence of hypersensitivity to the drug. *GRIA1* encodes a subunit of the AMPA receptor, a tetrameric ligand-gated ion channel that transmits glutamatergic signals in the brain. Glutamate not only has a role as a neurotransmitter, but also as an immunomodulator (Pacheco et al., 2007) and the study by Chen et al. (2011) provides the first link between *GRIA1* polymorphisms and an immune-related phenotype such as the occurrence of hypersensitivity to L-asparaginase. A later genome-wide study by the same group using the HapMap lymphoblastoid cell lines tested more than 2 million SNPs and identified the aspartate metabolic routes as the most likely candidate pathway for asparaginase sensitivity.

Finally, polymorphisms in other genes that mediate the antileukemic effect of asparaginase, such as the asparaginase synthetase gene (*ASNS*), the basic region leucine zipper activating transcription factor 5 (*ATF5*), or the argininosuccinate synthase 1 (*ASS1*), have been associated to lower event-free survival of ALL patients, albeit the authors reported no associations with adverse effects (Rousseau et al., 2011).

## CYCLOPHOSPHAMIDE

Cyclophosphamide, an oxazophosphorine, bifunctional DNA alkylating agent, is crucial in the treatment of most pediatric and adult malignancies, including ALL. Cyclophosphamide is bioactivated in the liver by various enzymes of the hepatic P450 system including CYP2A6, CYP2B6, CYP2C8, CYP2C9, CYP2C19, CYP3A4, and CYP3A5 (Cox, 1979; Parekh and Sladek, 1993; Dirven et al., 1994; Hayes and Pulford, 1995; Ludeman, 1999; Huang et al., 2000), whilst detoxification of active metabolites is mainly mediated by aldehyde dehydrogenases (ALDH1A1 and ALDH3A1; Parekh and Sladek, 1993) and glutathione S-transferases (GSTA1, GSTM1, GSTP1, and GSTT1; Hayes and Pulford, 1995). These enzymes are known to have functional polymorphisms, some of which have been found to impact toxicity associated with cyclophosphamide-based therapies in several pathologies (Zhong et al., 2006; Cho et al., 2010).

A study conducted in patients with leukemia who underwent hematopoietic stem cell transplantation showed that carriers of CYP2B6*2A or CYP2B6*4 variant alleles treated with cyclophosphamide were at higher risk of developing hemorrhagic cystitis and oral mucositis, respectively (Rocha et al., 2009). Interestingly, at least the CYP2B6*4 variant has been related to increased enzyme activity (Lang et al., 2001; Kirchheiner et al., 2003), and presumably this could lead to increased bioactivation of the drug and therefore could explain the observed higher incidence of toxicity in the patients. Numerous other *CYP2B6* alleles that have been associated with changes in the enzyme activity/expression (www.cypalleles.ki.se/cyp2b6.htm) and therefore they could be relevant with regard to cyclophosphamide-induced side effects. It should also be remarked that the *CYP2B6* gene exhibits a large intraethnic variability. Indeed, novel allelic variants and different linkage disequilibrium values have been described in certain populations (Restrepo et al., 2011), which should be taking into account when implementing tailored genotyping protocols aimed to determine the potential of *CYP2B6* as a biomarker of drug response.

Allan et al. (2001) have reported that the 105Val allele of the *GSTP1* Ile105Val polymorphism was overrepresented in a group of patients with therapy-related acute myeloid leukemia. Interestingly, this association was only observed in therapies including GSTP1 substrates such as cyclophosphamide. The authors hypothesized that an overexposure to the drug due to reduced detoxification capabilities could have played a role in leukemogenesis. Moreover, the same variation of the *GSTP1* gene has been suggested to be one of the factors determining a higher neurotoxicity of ifosfamide, a closely related agent (Zielinska et al., 2005). A later report showed that the presence of the 677C-1298C haplotype in the methylenetetrahydrofolate reductase (*MTHFR*) gene was also associated to secondary acute myeloid leukemia after cyclophosphamide treatment of hematologic malignancies (Guillem et al., 2007).

Other polymorphisms reportedly associated with increased cyclophosphamide-induced toxicity are CYP2C19*2 (Takada et al., 2004; Ngamjanyaporn et al., 2011), CYP3A4*1B (Su et al., 2010), *GSTM1/T1 *null (Cho et al., 2010), ALDH3A1*2 and ALDH1A1*2 (Ekhart et al., 2008), *ABCC4* rs9561778 (Low et al., 2009), or *ABCG2 Q141K *(rs2231142; Kim et al., 2008). It should be stated that some of these studies were conducted in patients with hematological malignancies but there are as yet no available data on ALL.

Cyclophosphamide is a chemotherapeutic agent used in a broad array of malignancies, which somewhat hampers the reproducibility of these findings. Therefore, larger studies and more consistent populations are needed in order to unequivocally establish the impact of the aforementioned SNPs and to identify other variants that could account for increased toxicity in cyclophosphamide-based therapies in ALL.

## CYTOSINE ARABINOSIDE

Cytosine arabinoside (Cytarabine, Ara-C) is an antimetabolite widely used in acute leukemia, which is associated with several adverse side effects, including myelosuppression, infections, mucositis, neurotoxicity, and acute pulmonary syndrome (Hartford et al., 2009). Candidate gene approaches have been used to identify genetic variables that are important in susceptibility to Ara-C. These studies have mainly focused on genes in the pharmacokinetic pathway of the drug. For instance, a common polymorphism, A79C (rs2072671), in the cytidine deaminase (*CDA*) gene, which catalyzes the rapid deamination of Ara-C, results in lower enzyme activity and hence a decreased rate of Ara-C metabolism (Kirch et al., 1998). Interestingly, Bhatla et al. (2009) have shown that Ara-C-related mortality was significantly elevated in carriers of the 79CC genotype in children with acute leukemia. Moreover, Ciccolini et al. (2012) have related this SNP to life-threatening toxicities induced in a girl with lymphoma treated with Ara-C.

Hartford et al. (2009) utilized an unbiased whole-genome approach to find polymorphisms that might predict the susceptibility to the cytotoxic effects of Ara-C in cell lines derived from persons of European (CEU) and African (YRI) ancestry. The authors identified a unique pharmacogenetic signature consisting of four SNPs explaining 51% of the variability in sensitivity to ara-C among the CEU and five SNPs explaining 58% of the variation among the YRI. These unique genetic signatures comprised novel target genes, most importantly *GIT1*, *RAD51AP1*, and *SLC25A37*, which can be studied further in functional studies. Furthermore, the authors examined 64 SNPs in the deoxycytidine kinase gene (*DCK*), which catalyzes the most essential step in the Ara-C activation pathway. Their conclusions show that cells that carried the *DCK* A70G (Ile24Val) polymorphism, which affects protein function, had an increased sensitivity to Ara-C (Hartford et al., 2009). In this regard, a later study has suggested that another *DCK* SNP, C-360G, is associated with the occurrence of mucositis after low-dose Ara-C in pediatric ALL patients (Banklau et al., 2010).

Recently, a study by Xu et al. (2012) seems to confirm the importance of both *CDA* and *DCK* genes as important loci that should be further investigated regarding the outcome of Ara-C-based chemotherapy in leukemia patients.

## CYTOTOXIC ANTIBIOTICS

Doxorubicin (adriamycin) and daunorubicin (daunomycin) are anthracycline antibiotics commonly used in the treatment of leukemias whose main concern is their well-known dose-related cardiotoxicity (Gilladoga et al., 1976).

The contribution of pharmacogenetic factors across the doxorubicin biochemical pathway is not well established, but the drug is characterized by inter-individual variation in pharmacokinetic and pharmacodynamic parameters, and genetic variation has been suggested to account for at least part of this variability (Jamieson and Boddy, 2010; Lal et al., 2010).**For instance, SNPs in the *ABCB1* and *SLC22A16* transporter genes have been shown to increase exposure levels that could result in a higher incidence of adverse effects (Lal et al., 2007, 2008). No studies on the impact of these mutations on the incidence of adverse effects in ALL patients have as yet been conducted. In patients of breast cancer treated with doxorubicin, Bray et al. (2010) have shown that several SNPs in the *SLC22A16* influx transporter gene (A146G, T312C, and T755C) are related to lower incidence of dose delay, indicative of less toxicity; although it should be noted that the authors considered their own findings as preliminary.

In addition, *in vitro* studies have shown that the wild-type allele of the Val88Ile (262G > A, rs1143663) SNP in the carbonyl reductase 1 (*CBR1*) gene, involved in doxorubicin metabolism, exhibits a higher rate of synthesis of cardiotoxic metabolites (Gonzalez-Covarrubias et al., 2007). The conclusions of this work seem to indicate that the small percentage of individuals of African ancestry (to which this SNP is confined) who are homozygous for the low-activity Ile88 allele would therefore be at lower risk of cardiotoxicity. Paradoxically, this would imply that a majority of the African population is at-risk for doxorubicin-induced toxicity. Interestingly enough, a study by Hasan et al. (2004) evaluated 100 African American patients who underwent doxorubicin-based combination therapy and found that they appeared to suffer cardiotoxicity from doxorubicin three times more frequently than previously studied populations. Because of the low sample size analyzed and the lack of head-to-head comparison, larger studies in a multiracial setting seem necessary to clarify this finding.

With regard to daunorubicin, a recent work has shown a trend toward significant increase in the drug systemic exposure in patients carrying the C-allele of the *CBR1* G312C (Leu73Leu, rs25678) polymorphism, which could elevate the risk of drug-induced toxicity (Varatharajan et al., 2012). In any case, it should be stated that the genetic associations reported for these two anthracyclines are still relatively recent and have not been consistently observed yet.

Mitoxantrone is an anthracycline analog which is a known substrate for ABC efflux transporters such as those encoded by the *ABCB1* and *ABCG2* genes (Kodaira et al., 2010). To date, there are no studies in the ALL setting testing the plausible hypothesis that genetic polymorphisms in these genes could affect mitoxantrone disposition and hence modulate the response and side effects to the drug. However, Cotte et al. (2009) have determined the frequencies of seven *ABCB1* and *ABCG2* SNPs in multiple sclerosis patients treated with mitoxantrone. Several associations were reported regarding the clinical response rate, but no relevant differences in genotype frequencies were observed in a subset of patients with severe hematological or cardiac side effects. However, it was intriguing that one patient presenting cardiomyopathy after a low dose of mitoxantrone was found to carry an uncommon genotype with homozygous variant alleles in two *ABCB1* (G2677T/A, rs2032582 and C3435T, rs1045642) and one *ABCG2* locus (Gln141Lys, rs2231142), in addition to a variant allele for the *ABCC2* C-24T (rs717620) SNP (Cotte et al., 2009).

## ETOPOSIDE

Etoposide is a topoisomerase II inhibitor used in a variety of malignancies. This drug is a substrate for the P-glycoprotein transporter, CYP3A4 and CYP3A5 isoforms (Relling et al., 1994), whose expression is partly regulated by the vitamin D receptor (VDR; Drocourt et al., 2002), and a number of phase II metabolizing enzymes, including GSTs and UGT1A1 (Watanabe et al., 2003). Therefore, alterations in these genes hold the potential to be relevant for the drug concentrations and clinical effects. However, to our knowledge there are as yet no studies investigating the association of polymorphisms in the etoposide pathways with the occurrence of adverse effects, mainly dose-limiting myelosuppression, in patients treated with the drug. Currently, there are only indications based on the impact of these genetic variations on the level of exposure to the drug. For instance, a study in 109 children diagnosed with ALL showed that carriers of both the CYP3A5*3/*3 and *GSTP1* Ile/Ile genotype displayed a lower drug clearance, although this association was only observed in African Americans one month after treatment (Kishi et al., 2004). Moreover, the *ABCB1* C3435T SNP was found to be an independent predictor of etoposide clearance disregarding ethnicity. In contrast, one year after treatment, the *UGT1A1* 6/6 (*1/*1), *VDR* intron 8 GG and *VDR* Fok 1 CC genotypes predicted higher clearance in African Americans (Kishi et al., 2004). Despite that the drug pharmacokinetics were shown to correlate with the incidence of adverse effects, no analyses of the association between SNPs and toxicity were conducted by the authors (Kishi et al., 2004).

Two genome-wide studies by an American research group have identified both genomic regions and SNPs associated with cellular sensitivity to etoposide. Huang et al. (2007) identified 63 genetic variants that contributed to etoposide-induced cytotoxicity through the evaluation of cell growth inhibition in cell lines from multi-generational pedigrees. The variants were present in genes whose expression had previously been related to altered cell sensitivity to etoposide, such as *AGPAT2*, *IL1B*, and *WNT5B*, but also in other genes not yet known to be associated with sensitivity to this agent. A limitation of this study was that candidate genes known to contribute to the pharmacokinetics of etoposide, e.g., *CYP3A*, *UGT1A1*, and *ABCB1*, are not expressed or are expressed at very low levels in the lymphoblastoid cell lines utilized.

Using the same cell models, Bleibel et al. (2009) later identified 22 unique SNPs in four genes among three chromosomes significantly associated with cytotoxic phenotypes at one or more treatment conditions. Genes implicated were *UVRAG*, a DNA repair gene, *SEMA5A*, which encodes semaphoring-5A protein involved in axonal guidance during development, the *SLC7A6* transporter gene, which participates in nitric oxide synthesis that ultimately induces apoptosis and *PRMT7*, encoding the protein arginine methyltransferase that catalyzes an irreversible protein modification. All these processes would be altered in the presence of functional polymorphisms and could presumably lead to the observed increased cytotoxicity in etoposide-treated cells.

Unfortunately, and despite the interesting background provided by these two genome-wide reports, there are as yet no clinical studies that have investigated the role of any of the aforementioned genetic variants in patients treated with etoposide.

## IMATINIB

Imatinib is a Bcr-Abl tyrosine kinase inhibitor that is specifically used in the treatment of Phy + leukemia. The drug is demethylated to *N*-desmethyl-imatinib by CYP3A4/5, with other CYP450 isoforms playing a less important role (Peng et al., 2005). Indeed, coadministration of inhibitors and inducers of CYP3A activity results in significant modifications of the drug’s pharmacokinetics (Bolton et al., 2004), which show a wide interindividual variability (Judson et al., 2005). In order to explain this variability, pharmacogenetics studies have been carried out to find genetic determinants that could modify the pharmacological response to the drug. Most of these studies have aimed to find genetic markers of resistance. Thus, a number of polymorphisms in genes coding for transporters (*ABCB1*, *ABCG2*, *SLC22A1*), drug metabolizing enzymes (*CYP3A5*), proteins with involved in the nucleotide excision repair pathway (*EERC*) and proteins related to leukemogenesis (*SOCS1 *and *PTPN22*) have been associated to the efficacy of the treatment (Dulucq and Krajinovic, 2010; Kong et al., 2012; Vivona et al., 2012). These studies have mainly been conducted in patients with chronic myeloid leukemia (CML) and gastrointestinal stromal tumors (GIST), probably because of the higher incidence of these two diseases.

On the other hand, pharmacogenetic studies on imatinib-related toxicity are scarce and therefore the clinical impact of genetic polymorphisms on the occurrence of adverse effects has to be inferred from their effect on the drug blood levels, as it has been reported that the severity of the side effects seems to correlate with the drug’s pharmacokinetics (Judson et al., 2005). In this regard, two studies have shown a reduced oral clearance of the drug in patients carrying the CYP2D6*4 (Gardner et al., 2006) or *ABCG2 421A* variant alleles (Petain et al., 2008), which could hypothetically result in increased adverse effects. Interestingly, Gurney et al. (2007) reported a higher drug clearance but also a less common toxicity-related dose reduction in CML patients on imatinib who were carriers of the TT genotype at each of three key positions in the *ABCB1* gene (1236, 2677, and 3435).

These data are not yet sufficiently conclusive to translate into individual drug dose adjustments and therefore further studies are still needed to analyze other genetic variants that can help individualize imatinib therapy.

## MERCAPTOPURINE

6-Mercaptopurine (6-MP) is an antimetabolite that has been used for 40 years in the treatment of a variety of diseases. In particular, 6-MP in orally daily regimen associated with weekly methotrexate (MTX) is the backbone of maintenance chemotherapy for ALL. The drug is metabolized toward active, and toxic, 6-thioguanine nucleotides (6-TGN) that are responsible for the elevated myelotoxicity of this chemotherapy agent.

**FIGURE 1:**
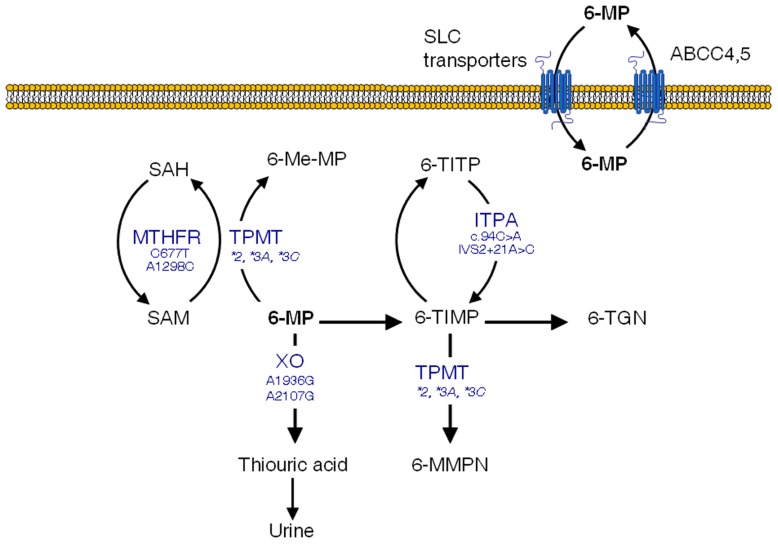
**6-mercaptopurine (6-MP) intracellular pathways with polymorphisms suggested to increase the occurrence of adverse effects**. SAM, S-adenosylmethionine; SAH, S-adenosylhomocysteine; TPMT, thiopurine S-methyltransferase; XO, xanthine oxidase; 6-Me-MP, 6-methyl-mercaptopurine; 6-TIMP, 6-thioinosine monophosphate; 6-TITP, 6-thioinosine triphosphate; ITPA, inosine triphosphate pyrophosphatase; 6-MMPN, 6-methyl-mercaptopurine nucleotides; 6-TGN, 6-thioguanine nucleotides

The thiopurine methyltransferase (*TPMT*) gene, codes for a key enzyme in the metabolism of 6-MP (**Figure 1**) and other related thiopurine drugs such as 6-thioguanine and azathioprine. This enzyme is affected by functional polymorphisms that have been shown to produce a defective enzyme, amongst them, TPMT*2 (G238C), TMPT*3A (G460A, A719G), and TPMT*3C (A719G) account for 90% of the enzymatic deficiency in most populations (Weinshilboum, 2001). Approximately one in 300 hundred individuals are homozygous for these variants and therefore lack TPMT activity, which results in high levels of 6-TGN and acute hematopoietic toxic effects, greater risk for radiation-induced brain tumors and a greater likelihood of chemotherapy-induced acute myeloid leukemia (Pui et al., 2004). Because of this, extreme caution should be exerted in those children with ALL who lack TPMT activity and are scheduled to receive 6-MP. In turn, these subjects are far less prone to experience a relapse (Lennard and Lilleyman, 1996). Dose reductions of up to 90% have been proved useful in these patients (McLeod et al., 2000). For heterozygous carriers the administration of 50% of the standard dose is recommended, although there has been a certain degree of controversy as to whether these patients with intermediate activity may benefit from lower doses (McLeod et al., 1999; Stanulla et al., 2005).

Having said that, the *TPMT* polymorphism does not explain all 6-MP-induced adverse effects, and some severe toxicities leading to life-threatening conditions remain unexplained (Palmieri et al., 2007). Additional SNPs in genes encoding enzymes involved in 6-MP metabolism and transport might contribute to the drug-induced toxicity. This is the case of the inosine triphosphate pyrophosphatase (ITPA) enzyme, which catalyzes one of the intermediate steps of 6-MP metabolism (**Figure 1**). Two SNPs in the *ITPA* gene with a frequency of roughly 10% in Caucasians (Adam de Beaumais and Jacqz-Aigrain, 2012), namely a non-synonymous C94A transition (rs1127354, Pro32Thr) and the intronic IVS2 + 21A > C mutation have been related with defective enzyme activity (Heller et al., 2004) leading to higher risk of myelotoxicity and hepatotoxicity in ALL pediatric patients (Hawwa et al., 2008; Stocco et al., 2009; Wan Rosalina et al., 2011). In addition, *MTHFR* SNPs have been shown to be more common in subjects with low TPMT activity (Karas-Kuzelicki et al., 2009), probably because of their impact on S-adenosylmethionine, which functions as a cofactor for TPMT. Other polymorphisms in genes involved in 6-MP disposition such as xanthine oxidase or *ABCC4* have also been suggested to impact clinical outcomes, although there are as yet no available data in ALL patients (Hawwa et al., 2008; Ban et al., 2010).

The use of the TPMT genotyping for tailoring ALL therapy, and the putative inclusion of other polymorphisms in these genetic tests will be discussed in the final chapter of this review.

## METHOTREXATE

Methotrexate (MTX) is a folate inhibitor widely employed in the chemotherapy of hematologic malignancies and various solid tumors. This drug is the cornerstone for therapy of ALL and has been the focus of a number of pharmacogenetic studies aimed to identify genetic determinants of its toxicity (reviewed elsewhere Gervasini, 2009). Its actions on folate metabolism follow a complex pattern that includes several transporters and metabolizing enzymes whose function and/or expression have been suggested to be altered by genetic polymorphisms.

**FIGURE 2:**
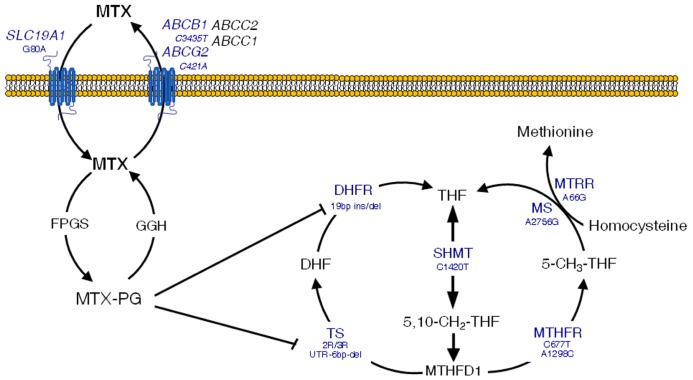
**Overview of methotrexate (MTX) mechanisms of action in the folate metabolic pathway**. Clinically significant polymorphisms are shown for the main genes involved. MTX-PG, methotrexate polyglutamates; *SLC19A1*, solute carrier 19A1; *ABCC1* and* 2*, ATP-binding cassette C1 and 2 transporters; *ABCG2*, ATP-binding cassette G2 transporter, breast cancer resistance protein; *ABCB1*, ATP-binding cassette B1 transporter, multidrug resistance; FPGS, folylpolyglutamyl synthase; GGH, γ-glutamyl hydrolase; *TS*, thymidylate synthase; UTR, untranslated region; *DHFR*, dihydrofolate reductase; *SHMT*, serine hydroxymethyltransferase; *MTHFD1*, methylenetetrahydrofolate dehydrogenase; *CCND1*, cyclin D1; *ATIC*, aminoimidazole 4-carboxamide ribonucleotide (AICAR) transformylase; *MS*, methionine synthase; *MTRR*, methionine synthase reductase. Adapted with permission from Gervasini (2009).

Briefly, the reduced folate carrier 1 (*RFC1, SLC19A1*) is responsible for the entry of MTX in the cell (Moscow et al., 1995), whilst the drug is pumped out by a variety of ATP-binding cassette (ABC) efflux transporters (Strand et al., 1999). In the cell, MTX is metabolized to active polyglutamates, which are responsible for the disruption of the folate metabolic pathway by inhibiting enzymes that are essential for the DNA (Chabner et al., 1985). These include thymidylate synthase (TS; Szeto et al., 1979) and dihydrofolate reductase (DHFR; Galivan, 1980). Another key enzyme in the folate pathways is methylenetetrahydrofolate reductase (MTHFR), which produces 5-methyl-tetrahydrofolate (THF) from 5,10-methylene-THF, a major intermediary that is in turn synthesized by serine hydroxymethyltransferase (SHMT1). Finally, the production of 5-methyl-THF is pivotal for biotransformation of homocysteine to methionine, which involves two major enzymes, namely methionine synthase (MS) and methionine synthase reductase (MTRR). **Figure 2** depicts an overview of these intracellular pathways pointing out those SNPs that have been related with the occurrence of MTX-induced toxicity.

*MTHFR* has by far been the most extensively studied gene in association studies in ALL. Two SNPs, C677T (rs1801133) and A1298C (rs1801131), have been related to increased MTX toxicity (Kantar et al., 2009; D’Angelo et al., 2011). However, a recent meta-analysis (Yang et al., 2012), as well as the general perception in the literature (reviewed by Gervasini, 2009) lead to think that C677T is the only SNP that plays a clinically significant role, albeit contradictory results also exist (Chatzidakis et al., 2006; Kishi et al., 2007; Huang et al., 2008).

Most notably, given the severity of the syndrome, the 677T allele has been suggested to induce MTX-related neurotoxicity (mainly subacute leukoencephalopathy) in young ALL patients (Strunk et al., 2003; Mahadeo et al., 2010; Vagace et al., 2011, 2012). It should be noted that this association has only been observed in clinical case reports. In fact, a study in 53 children with ALL treated with high-dose MTX failed to confirm a relevant role of the 677T allele in the drug-induced neurotoxicity developed by nine of the patients (Kishi et al., 2003). In order to elucidate this controversy, it would be desirable to genotype large populations of pediatric ALL patients who underwent MTX-related neurotoxicity. However, the lack of homogeneity of treatment protocols, which makes multicenter studies hard to conduct, the low number of patients affected, and the more than likely influence of other SNPs, have so far been insurmountable obstacles.

A 19 bp ins/del polymorphism in the *DHFR* gene has also been associated with increased toxicity in adult leukemia patients treated with MTX (Ongaro et al., 2009). In the same manner, the *MTRR* A66G SNP has been related to increased risk of developing toxicity in children with ALL treated with high doses of MTX (Huang et al., 2008), although there seems to be no relation with CNS side effects (Krajinovic et al., 2005).

Other genes that could also be involved in the development of MTX-induced toxicity are the ABC efflux transporters. Their location in the blood-brain barrier makes tempting to speculate that polymorphisms in these genes could lead to drug accumulation in the brain and subsequent neurotoxicity. For instance, the combined presence of the C421A (rs2231142, Gln141Lys) SNP in the *ABCG2* gene (encoding the Breast Resistance Cancer Protein, BCRP transporter) and the C3435T transition in *ABCB1* (coding for P-glycoprotein) has been related to the occurrence of encephalopathy in children with ALL treated with MTX (Erdilyi et al., 2008). Finally, the G-80A polymorphism in the RFC1**influx transporter, which determines intracellular levels of MTX, has been associated with increased overall toxicity in ALL patients (Shimasaki et al., 2006; Imanishi et al., 2007; Kishi et al., 2007).

It should be noted that large studies on the association between MTX toxicity and genetics in childhood ALL are still scarce and mostly focus on *MTHFR* variants. Studies carried out in other pathologies have found evidences of increased toxicity in the presence of variant alleles in additional ABC transporters (Campalani et al., 2007; Ranganathan et al., 2008), or in the *SHMT1* (Weisman et al., 2006) and *TS* genes (Campalani et al., 2007). Therefore, we should not rule out the implication of these polymorphisms in MXT-related side effects experienced by ALL patients.

## VINCRISTINE

The wide pharmacokinetic interindividual variability shown by vincristine, and the occurrence of its dose-limiting neurotoxicity remains largely unpredictable (Moore and Pinkerton, 2009). Renbarger et al. (2008) have showed different grades of neurotoxicity in Caucasians compared to African Americans, suggesting that genetics could influence vincristine-induced toxicity.

The main candidate gene for pharmacogenetic studies is *CYP3A5*, since it is known to contribute between 55 and 95% to total vincristine metabolism (Dennison et al., 2006). In addition, the drug is a substrate for P-glycoprotein (Song et al., 1999), which makes the encoding *ABCB1* gene another suitable candidate for pharmacogenetic studies. Two reports have evaluated the clinical effect of common SNPs (CYP3A5*3 and *ABCB1* C3435T and G2677T) in these genes in pediatric ALL patients treated with vincristine. However, the authors failed to find a significant association with increased occurrence of side effects such as impaired motor activity or constipation (Plasschaert et al., 2004; Hartman et al., 2010).These two studies combined added up to only 86 genotyped patients, which is clearly not sufficient to rule out the involvement of these candidate genes in vincristine toxicity.

## PERSPECTIVES AND CONCLUSION

The utility of pharmacogenetics in clinical routine has turned to be lower than anticipated, as numerous barriers to implementing individualized medicine have appeared over the years (Agundez et al., 2012). Cases in which a genetic test is sufficient to significantly affect a given therapy are discouragingly uncommon (Groenen, 2011) and the leukemia setting is not an exception. Indeed, only 1–2% of marketed drugs have pharmacogenomic-based recommendations (Agundez et al., 2012) and amongst the drugs commonly used in the chemotherapy of pediatric ALL, only 6-MP, thioguanine and azathioprine labels include genetic testing (*TPMT*) as a recommendation to help individualize therapy. However, steps are being taken in order to revert this situation. The increasing availability of low-cost, high-throughput genetic platforms that allow the simultaneous screening of hundreds of polymorphisms, the education of health practitioners or the implementation of multicenter networks aimed to improve the safety of new drugs, are some of the measures that hopefully will help improve the impact of pharmacogenetics on clinical routine. It should also be pointed out that genetic variability in several of the targets for which genetic testing is recommended by the FDA and other corporations has been shown to modify the risk of acute leukemia (Agundez, 2004), which certainly enhances the utility of these tests.

The body of work on the clinical impact of *TPMT* and *MTHFR* polymorphisms on ALL does not mirror the limited current knowledge on the pharmacogenetics of the other drugs that constitute the core of chemotherapy for this disease. Thus, significantly less information is available with regard to vincristine, asparaginase, cyclophosphamide etc. The *TPMT* gene polymorphism provides the best example of the value of applied pharmacogenetics in ALL and clinical oncology in general. It is now widely acknowledged that the initial dose of 6-MP treatment should be based on the *TPMT* genotype (Relling et al., 2011), thus allowing the clinician to identify patients at higher risk of toxicity. However, as it was described in the 6-MP chapter, there is still some interindividual variability in the response to this drug that cannot be explained only by the *TPMT* genotype (Palmieri et al., 2007). Therefore, the implementation of additional genetic analyses to identify polymorphisms in the *ITPA*, *MTHFR*, *XO* and other genes in the 6-MP intracellular pathway, as well as the study of their epistatic interactions seem to be reasonable steps to take in order to better adjust 6-MP doses in ALL patients. Furthermore, pharmacogenomics alone may not be sufficient to explain all the interindividual variability in 6-MP response and efforts should be undertaken in the coming years to create more precise algorithms that can help predict drug response.

With regard to *MTHFR*, the reasons for the present controversy regarding the influence of its genetic variation on MTX-induced toxicity are diverse. For instance, the wide variety of diseases in which MTX has been proved useful has paradoxically hampered the reproducibility of the results of genetic association studies, because of the heterogeneity of the patients analyzed. Moreover, other unknown variants in the same gene, epistatic interactions with other genes and, most likely, the combination of these factors, may result in genetic backgrounds with different susceptibilities to MTX-induced toxicity. The challenge seems to be to identify which genetic factors and genetic combinations are those in specific populations.

Some ALL chemotherapy regimens contemplate MTX dose reductions for subjects homozygous for the 677T variant or for those carrying both 677CT and 1298AC heterozygous genotypes. One could consider at least premature to make this kind of dose adjustments based on data that are still controversial. In fact, MTX was not included in the list of drugs mentioned in the *Table of Pharmacogenomic Biomarkers in Drug Label* issued by the Food and Drug Administration (FDA) last year (FDA, 2011)*. *It is probably naive to believe that the determination of SNPs in just one gene (*MTHFR*) belonging to a highly complex intracellular pathway, such as that of MTX, is enough to accurately anticipate the occurrence of adverse effects.

As a general rule, it would be logical to think that the identification of combinations of mutations in several genes along the pathway of a given drug must be more helpful in terms of identifying subjects at-risk of toxicity than a single-SNP approach (Vagace et al., 2011). This is crucial in the case of drugs with intricate intracellular routes as it is the case of MTX or 6-MP. Genome-wide approaches such as the ones reported by (Huang et al., 2007), Bleibel et al. (2009), and Chen et al. (2011) are able to evaluate whole pathways to identify key routes that can be later studied in detail. In addition, the inclusion of other clinical and demographic factors may increase the predictive value of pharmacogenetic models (Wessels et al., 2007).

## Conflict of Interest Statement

The authors declare that the research was conducted in the absence of any commercial or financial relationships that could be construed as a potential conflict of interest.
